# The accuracy and consistency of mastery for each content domain using the Rasch and deterministic inputs, noisy “and” gate diagnostic classification models: a simulation study and a real-world analysis using data from the Korean Medical Licensing Examination

**DOI:** 10.3352/jeehp.2021.18.15

**Published:** 2021-07-05

**Authors:** Dong Gi Seo, Jae Kum Kim

**Affiliations:** 1 Department of Psychology, College of Social Science, Hallym University, Chuncheon, Korea; 2 Hallym Applied Psychology Institute, College of Social Science, Hallym University, Chuncheon, Korea; 3 Korea International University in Ferghana, Ferghana, Uzbekistan; Hallym University, Korea

**Keywords:** Data collection, Data analysis, Psychometrics, Republic of Korea, Statistical models

## Abstract

**Purpose:**

Diagnostic classification models (DCMs) were developed to identify the mastery or non-mastery of the attributes required for solving test items, but their application has been limited to very low-level attributes, and the accuracy and consistency of high-level attributes using DCMs have rarely been reported compared with classical test theory (CTT) and item response theory models. This paper compared the accuracy of high-level attribute mastery between deterministic inputs, noisy “and” gate (DINA) and Rasch models, along with sub-scores based on CTT.

**Methods:**

First, a simulation study explored the effects of attribute length (number of items per attribute) and the correlations among attributes with respect to the accuracy of mastery. Second, a real-data study examined model and item fit and investigated the consistency of mastery for each attribute among the 3 models using the 2017 Korean Medical Licensing Examination with 360 items.

**Results:**

Accuracy of mastery increased with a higher number of items measuring each attribute across all conditions. The DINA model was more accurate than the CTT and Rasch models for attributes with high correlations (>0.5) and few items. In the real-data analysis, the DINA and Rasch models generally showed better item fits and appropriate model fit. The consistency of mastery between the Rasch and DINA models ranged from 0.541 to 0.633 and the correlations of person attribute scores between the Rasch and DINA models ranged from 0.579 to 0.786.

**Conclusion:**

Although all 3 models provide a mastery decision for each examinee, the individual mastery profile using the DINA model provides more accurate decisions for attributes with high correlations than the CTT and Rasch models. The DINA model can also be directly applied to tests with complex structures, unlike the CTT and Rasch models, and it provides different diagnostic information from the CTT and Rasch models.

## Introduction

### Background/rationale

One purpose of medical licensing examinations is to categorize students into performance or achievement levels for legal accountability requirements. This is done by assigning a student to a performance level based on his/her overall scaled score. However, educators often want diagnostic information about how a given student did on each content area in licensing examinations. This is often done by providing raw scores or percent correct scores for each content strand. Although popular among educators, psychometricians are leery of providing such scores. As an alternative, diagnostic strand scores can be provided by using item response theory (IRT) or the Rasch model. The Rasch model is useful for scaling students on single or multiple latent proficiencies based on a simple structure [1]. Thus, the Rasch model can be used to classify latent abilities with respect to attributes [2]. The Rasch model is expressed as:

(Equation 1) p(uijθj,bi)=eθj-bi1+eθj-bi

where *b_i_* is the difficulty estimate for item *i*, and *θ_j_* is the estimate of the ability of examinee *j*. The Rasch model assumes that the attributes of examinees are independent from each other.

However, in light of the above considerations, it is important to keep in mind that IRT and the Rasch model are used to scale the overall test and do not provide specific diagnostic information for each content domain. In contrast, diagnostic classification models (DCMs) have the specific purpose of identifying examinees who are masters or non-masters of each content strand. The deterministic inputs, noisy “and” gate (DINA) model is known to be a simple and efficient DCM [3]. The item response function in the DINA model is given by

(Equation 2) P(Xij=1ηij)=gi(1-ηij)(1-si)ηij

where *Х_ij_* identifies the response of examinee *j* to item *i* (where *i*=1,…,*i*) with 1 or 0 reflecting a correct or incorrect response, and denote the guess and slip parameters for the item *i*, respectively, and *η_ij_* is a binary indicator given by

(Equation 3) $$\eta_{ij}=\prod_{k=1}^{K}{\left(\alpha_{jk}\right)}^{q_{ik}}$$

which denotes whether examinee *j* has mastered all attributes assigned by item *i*. *α_jk_* is mastery of the *k^th^* attribute in the *j^th^* examinee, which is either 1 or 0 for *k*. *q_ik_* denotes an entry in the *i^th^* row, *k^th^* column of the matrix *Q*, mapping the attribute and item with the matrix *i* × *k*, for which individual entries take values from

(Equation 4) p(uijθj,bi)=eθj-bi1+eθj-bi

DCMs have become popular in educational evaluation. DCMs characterize examinees’ attributes for each content area using categorical latent variables that measure the skill/knowledge states of examinees [4]. Most DCMs utilize 2-category latent classes, with examinees being considered masters or non-masters of an attribute. An examinee is classified based on the probabilities at each categorical level of the latent attribute (i.e., the probabilities of mastery for 2-category attributes). Many studies on DCMs have estimated item parameters [5], analyzed model fit [6], and used DCMs in testing programs and research applications [7,8].

Although DCMs were developed to identify examinees’ mastery or non-mastery of attributes required for solving test items, their application has been limited to very low-level attributes (e.g., management, assessment, pathophysiology), few studies have reported the classification accuracy and consistency of DCMs for high-level attributes (e.g., cardiology, trauma, obstetric, pediatric and operations), which are of greater interest for educators. In addition, no study has empirically explored the relationship between IRT models and DCMs for high-stakes assessments.

### Objectives

This paper compared the accuracy and consistency of diagnostic skill reporting (students’ strengths and weaknesses in terms of mastery of content strands) between DINA and IRT/CTT models. In order to compare the sub-scores among 3 models, a simulation study was conducted to examine the effects of attribute size (number of items per attribute) and the correlations among the attributes. A real-data study was also carried out using a large-scale assessment. The simulation explored the accuracy of mastery or non-mastery among the 3 models, while the real-data study examined the models’ consistency of determining mastery or non-mastery of strands.

## Methods

### Ethics statement

The Korea Health Professional Licensing Examination Institute provided the raw data for research purposes. This open data source does not contain identification and personal information about the examinees. Therefore, the requirements for informed consent and institutional review board approval were exempted according to the Enforcement Rule of Bioethics and Safety Act of South Korea.

### Simulation study

Haberman and Sinharay [9] demonstrated the appropriateness of reporting sub-scores using multidimensional item response theory (MIRT) in large-scale assessments. Thus, a MIRT model was used to generate responses with items only measuring a single latent variable (simple structure), and those responses were then used to measure unbiased estimates for the CTT, DINA, and Rasch models. The following MIRT model was applied to generate responses [10]:

(Equation 5) p(uijθj,bi)=e∑k=1maikθjk-bi1+e∑k=1maikθjk-bi

where *b_i_* is the difficulty parameter of item *i*, and *θ_j_* is the latent attribute for examinee *j*. The value of *a_ik_* was set to 1 for all items in attribute *k*.

True difficulty values were generated from uniform distributions ranging from -3 to 3. Fifty test forms were replicated with 50 items per form (each strand contained 5, 10, 15, and 20 items, respectively). True *θ* values were drawn from a multivariate normal distributions with correlations of 0, 0.3, 0.5, 0.7, and 0.9 among 4 strands for each of 1,000 simulated examinees. For each simulated data set, the latent classes of each strand using the CTT, DINA, and Rasch models were classified using R code developed by the author [11]. The item parameters were estimated by the marginal maximum likelihood, and person parameters were computed by the maximum a posteriori method for the DINA and Rasch models [12]. This simulation study considered k=4 content strands (high-level attributes), implying the existence of L=2^4^=16 possible attribute mastery latent classes. Examinees were classified into classes based on the largest posterior probability (maximum a posteriori) for the DINA model. An individual examinee was classified as a master if each attribute’s *θ* value was higher than 0.0 in the Rasch model, and if the percent of correct scores was above 50% for each content strand in the CTT model. The Q-matrix for the DINA model was constructed as a simple structure with each item measuring only 1 attribute to create a parallel condition with the Rasch model, as shown in Table 1.

### Real-data study

The classification consistency and item fit indices were compared between the DINA and Rasch models using data from the 2017 Korean Medical Licensing Examination (KMLE), which consisted of 360 multiple-choice test items spread across 8 content strands (i.e., high-level attributes). The names and the number of items in each content strand was provided in Table 2. Mastery of each content strand was computed using the CTT, DINA, and Rasch models. As in the simulation study, an individual examinee was classified as a master if each attribute’s θ value was higher than 0.0 in the Rasch model, and if the percent of correct scores was above 0.5 for each content strand in the CTT model. In total, 3,265 examinees were analyzed in this study, using results that are available from Dataset 1 [8]. The Q matrix of the real data was constructed that each item required only one attribute, which showed the relationship between items and each content domain. The full Q matrix is available in Dataset 2.

### Accuracy and consistency of mastery

The accuracy index was used to measure the concordance between true and observed classifications. Since there are 2 mastery levels (true and observed mastery), a 2×2 contingency table between the 2 types of classifications for each attribute was created. The true mastery and observed mastery of the accuracy table represented the estimated proportion of students who had performance mastery based on their true score and whose observed score was classified as showing performance mastery using the CTT, DINA, and Rasch models. Accuracy of mastery in the simulation study was calculated as the proportion of students whose true and observed achievement levels matched one another, as computed by the sum of the diagonal elements of the accuracy table divided by the number of examinees. The consistency of mastery for real data was likewise computed using a contingency table presenting the proportion of students classified as exactly matching by 2 paired models (CTT versus Rasch, CTT versus DINA, Rasch versus DINA).

### Item-fit indices

Two different types of residuals are calculated in tests of fit of items to the Rasch model. Response residuals compare observed and expected values for every combination of person and item. The outfit index places equal weight on examinees’ ability when computing the fit index and is strongly affected by unexpected responses beyond the person ability measures. The outfit index has an expected value of 1.0, and ranges from 0 to positive infinity. A fit index greater than 1.0 indicates underfit and a fit index less than 1.0 indicates overfit to the Rasch model. This study considered values between 0.6 and 1.4 as acceptable [13]. The outfit index formulas are described in Equation 6 as the fit index in the Rasch model.

(Equation 6) $$Outfit=\frac{\sum_{j=1}^{N}\frac{{\left(X_{ij}-P_{ij}\right)}^{2}}{P_{ij}(1-P_{ij})}}{N}$$

where, *Х_ij_* is the response of examinee *j* to item *i* (where *i*=1,…,*i*) with 1 or 0 reflecting a correct or incorrect response, is the item response function for person *j* to item *i*, and *N* is the number of people. The root mean square error of approximation (RMSEA) of each item was used as the fit index for the DINA model. RMSEA values of 0.08 or lower were considered acceptable [14].

## Results

### Simulation Study

The results of the simulation study are presented in Table 3. The accuracy of mastery was slightly different between the Rasch and DINA models for all conditions, although both models were better than the CTT model. In the Rasch and DINA models, the accuracy of mastery decreased as the correlation among attributes increased from 0 to 0.5, and then gradually increased as the correlation among attributes increased from 0.5 to 0.9 with attribute sizes of 5, 10, and 15 items. Specifically, the DINA model was better than the Rasch and CTT models if there were high correlations among attribute, while the Rasch model was better than the DINA and CTT models if there were low correlations among attributes. The Rasch and DINA models were less accurate if the correlation among attributes was 0.5. This result was expected because the Rasch and DINA models assume independence among attributes. In CTT models, the accuracy of mastery consistently decreased as the correlation among attributes increased from 0 to 0.9. The accuracy of mastery increased as the content size increased from 5 items (strand 1) to 20 items (strand 4) in the CTT, Rasch, and DINA models. The DINA model was more accurate in terms of mastery than the Rasch and CTT models for attributes with a small amount of content, while the Rasch model was more accurate than the DINA and CTT models for medium and large amounts of content.

### Real-data study

#### Model fit

A unidimensional latent ability was assumed for the 3 models in this study, reflecting the fact that the KMLE was developed as a unidimensional assessment. An exploratory DETECT analysis was conducted using the expl.detect function in the “sirt” package [15] in R program [11]. The DETECT value was less than 0.2. Thus, the KMLE can be considered as essentially unidimensional [16]. Although the data were developed for a different purpose, the model fit indices based on the deviance (-2LL) were 999,063 for the Rasch model and 997,006 for the DINA model. The DINA model slightly fit better than the Rasch model with respect to diagnostic assessment of each examinee’s skill because the Rasch model is more parsimonious than the DINA model in that DIA model required content specification (all guessing and slipping parameters and the RMSEA in the DINA model are available in Dataset 3).

#### Item fit

The number of poor-fit items for both the Rasch and DINA models is described in Table 4. The criterion of poor-fit items for Rasch model was the outfit index, and the criterion used for the DINA model was the RMSEA (CTT does not provide model fit). In total, 5 items were flagged for the Rasch model and 10 items were flagged for the DINA model. Most flagged items were in content strand 5 due to its large amount of content. However, the flagged items were different between the Rasch and DINA models.

#### Consistency of mastery

Table 5 shows the consistency of mastery of 8 content domains among the CTT, Rasch, and DINA models. The consistency of mastery between Rasch and DINA models ranged from 0.541 to 0.610. The CTT and Rasch models were almost fully consistent, whereas the consistency between the CTT and DINA models was different from the consistency between the CTT and Rasch, but similar to the consistency between the Rasch and DINA models. An interesting finding is that the consistency of mastery between the CTT and DINA models was better than the consistency of mastery between the CTT and Rasch models in short content areas. Therefore, this study shows that the DINA model provides a different diagnostic perspective from those of the CTT and Rasch models.

#### Correlations of person estimates

Table 6 shows the correlations of person estimates for 8 content domains using the CTT, Rasch and DINA models. Across the 3 pairwise comparisons, the correlations of person estimates between CTT and Rasch were close to 1. The correlations of person attributes between the Rasch and DINA models ranged from 0.579 to 0.717. The correlations of person estimates between the CTT and DINA models were slightly higher than the correlations of person estimates between the CTT and Rasch models (R code for all computations are available in Dataset 4).

## Discussion

### Key results

It has become increasingly common to provide diagnostic information for educational and psychological assessments, and many studies have presented diagnostic information obtained using the CTT, Rasch, and DINA models. However, few studies have compared the accuracy and consistency of mastery among models. Therefore, the simulation study presented herein investigated the accuracy of mastery decisions made using the CTT, Rasch, and DINA models under varying assessment conditions. The results showed that the accuracy of mastery changed depending on the number of attributes and correlations among attributes. The DINA model worked better than the Rasch and CTT models for small attributes (5 items each) and attributes with a high correlation (>0.7). Then, real-world KMLE data were used to compare consistency among models. The consistency between the CTT and Rasch models was high, but lower consistency was found between the CTT and DINA models and between the DINA and Rasch models. Based on the initial simulation study, CTT had the lowest accuracy, meaning that the CTT and Rasch models were equally poor in the classification compared with the DINA model for real-world KMLE data.

### Interpretation

By applying the DINA model, educators can make low-level or high-level diagnostic inferences unlike those obtained using the CTT and Rasch models. Both analyses in this study demonstrated that the DINA model provides different perspective diagnostic information for each content area and performs well for short tests with high correlations among attributes. In general, the DINA model provides direct diagnostic information with respect to each content attribute for all examinees. In contrast, the Rasch model and CTT indirectly estimate the mastery of each content attribute by computing the probabilities of being a master for each attribute. Therefore, the DINA model provided more efficient and accurate diagnostic information than the CTT and Rasch models in a real-world high-stakes assessment. In addition, the DINA model provided similar model fit and item fit using KMLE data, even though the KMLE was not constructed for diagnostic purposes. Thus, the DINA model will work better if a practical assessment is constructed for diagnostic purposes or if the content domains are closely related to each other with a small number of items.

### Limitations

The simulation study and real-data study for the 3 models were applied for a test with a simple structure, which is a very restrictive model for real multi-dimensional data. In the practical setting, each item may be assigned to 2 or more content areas, corresponding to a complex structure model. For practical purposes, the DINA model was developed to estimate models with complex structures, rather than those with simple structures. It is known that DCMs are more suitable for complex relationships between items and attributes than the Rasch and CTT models. Since the KMLE data were obtained for a retrospective study, a limitation of this study is that it only dealt with a simple-structure model. Therefore, a more complex structure reflecting compensatory and non-compensatory models would be useful for analyzing DCMs in a future study. In addition, this study used only the DINA model as a single type of DCM for comparison with the Rasch and CTT models. To generalize the results further, it would be helpful to analyze several types of DCMs, such as the DINO (deterministic input noisy output “OR” gate) model.

### Conclusion

Despite the limitations of the current study, the DINA model worked well for providing diagnostic information in terms of mastery for each content area compared with the Rasch and CTT models. Specifically, the DINA model worked well in conditions with high correlations among attributes and attributes with a small number of items. Based on the findings of this study, the DINA model can be used for more efficient and complex diagnostic purposes in content mastery decisions instead of the Rasch and CTT models. In addition, DCM analysis would allow students to prepare for medical licensing examinations by identifying their strengths and weaknesses for improvement, thereby enhancing learning.

## Figures and Tables

**Table 1.** Q-matrix for the simulation data

Item	Attributes			
	A1	A2	A3	A4
1	1	0	0	0
2	1	0	0	0
3	1	0	0	0
4	1	0	0	0
5	1	0	0	0
6	0	1	0	0
7	0	1	0	0
8	0	1	0	0
9	0	1	0	0
10	0	1	0	0
11	0	1	0	0
12	0	1	0	0
13	0	1	0	0
14	0	1	0	0
15	0	1	0	0
16	0	0	1	0
17	0	0	1	0
18	0	0	1	0
19	0	0	1	0
20	0	0	1	0
21	0	0	1	0
22	0	0	1	0
23	0	0	1	0
24	0	0	1	0
25	0	0	1	0
26	0	0	1	0
27	0	0	1	0
28	0	0	1	0
29	0	0	1	0
30	0	0	1	0
31	0	0	0	1
32	0	0	0	1
33	0	0	0	1
34	0	0	0	1
35	0	0	0	1
36	0	0	0	1
37	0	0	0	1
38	0	0	0	1
39	0	0	0	1
40	0	0	0	1
41	0	0	0	1
42	0	0	0	1
43	0	0	0	1
44	0	0	0	1
45	0	0	0	1
46	0	0	0	1
47	0	0	0	1
48	0	0	0	1
49	0	0	0	1
50	0	0	0	1

**Table 2.** Item information across 8 content domains

Content domains	No. of items (%)
C1	45 (12.50)
C2	45 (12.50)
C3	45 (12.50)
C4	25 (6.94)
C5	154 (42.78)
C6	20 (5.56)
C7	20 (5.56)
C8	6 (1.67)
Total	360 (100.00)

**Table 3.** Classification accuracy of the CTT, Rasch, and DINA models

Attribute size	Model	Correlation between attributes				
		0	0.3	0.5	0.7	0.9
5 items	Rasch	0.728	0.730	0.706	0.728	0.753
	DINA	0.728	0.738	0.717	0.738	0.753
	CTT	0.632	0.623	0.621	0.614	0.611
10 items	Rasch	0.785	0.779	0.784	0.786	0.788
	DINA	0.764	0.778	0.784	0.789	0.789
	CTT	0.723	0.721	0.712	0.711	0.709
15 items	Rasch	0.813	0.821	0.812	0.814	0.821
	DINA	0.795	0.814	0.800	0.820	0.823
	CTT	0.744	0.732	0.721	0.712	0.709
20 items	Rasch	0.850	0.838	0.820	0.827	0.830
	DINA	0.819	0.820	0.819	0.832	0.836
	CTT	0.766	0.755	0.743	0.742	0.731

CTT, classical test theory; DINA, deterministic inputs, noisy “and” gate.

**Table 4.** The number of flagged items in the 8 content domains in the Rasch and DINA models

Content domains	No. of items	# of flagged items in Rasch	# of flagged items in DINA
C1	45	0	0
C2	45	1	0
C3	45	1	0
C4	25	0	0
C5	154	3	10
C6	20	0	0
C7	20	0	0
C8	6	0	0
Total	360	5	10

DINA, deterministic inputs, noisy “and” gate.

**Table 5.** Consistency of mastery for person estimates for 8 content domains using the CTT, Rasch, and DINA models

Content domains	CTT and Rasch	CTT and DINA	Rasch and DINA
C1	1	0.610	0.610
C2	0.988	0.625	0.614
C3	1	0.597	0.597
C4	0.965	0.581	0.547
C5	0.997	0.601	0.603
C6	1	0.594	0.594
C7	0.993	0.639	0.633
C8	0.469	0.720	0.541

CTT, classical test theory; DINA, deterministic inputs, noisy “and” gate.

**Table 6.** Correlations of person estimates for 8 content domains using the CTT, Rasch, and DINA models

Content domains	CTT and Rasch	CTT and DINA	Rasch and DINA
C1	0.988	0.739	0.717
C2	0.994	0.761	0.755
C3	0.984	0.728	0.717
C4	0.991	0.688	0.682
C5	0.990	0.796	0.786
C6	0.993	0.609	0.594
C7	0.997	0.662	0.654
C8	1	0.579	0.579

CTT, classical test theory; DINA, deterministic inputs, noisy “and” gate.

## Footnotes

**Data availability**
Data files are available from Harvard Dataverse: https://doi.org/10.7910/DVN/XNKYZS Dataset 1. Raw data files of Korea medical licensing examination are available from: https://doi.org/10.7910/DVN/PETWZF. Dataset 2. Q matrix data.

Dataset 3. Item parameters data including slipping and guessing in DINA.

Dataset 4. R code for KMLE real data study.

## Supplementary materials

Supplement 1. Audio recording of the abstract.

## References

[1] Rasch G (1993). Probabilistic models for some intelligence and attainment tests.

[2] De Ayala RJ (2009). The theory and practice of item response theory.

[3] Junker BW, Sijtsma K (2001). Cognitive assessment models with few assumptions, and connections with nonparametric item response theory. Appl Psychol Meas.

[4] Rupp AA, Templin J, Henson RA (2010). Diagnostic measurement: theory, methods, and applications.

[5] De La Torre J (2009). DINA model and parameter estimation: a didactic. J Educ Behav Stat.

[6] De La Torre J, Douglas JA (2008). Model evaluation and multiple strategies in cognitive diagnosis: an analysis of fraction subtraction data. Psychometrika.

[7] De La Torre J, Douglas JA (2004). Higher-order latent trait models for cognitive diagnosis. Psychometrika.

[8] Seo DG, Choi J (2018). Post-hoc simulation study of computerized adaptive testing for the Korean Medical Licensing Examination. J Educ Eval Health Prof.

[9] Haberman SJ, Sinharay S (2010). Reporting of subscores using multidimensional item response theory. Psychometrika.

[10] Reckase MD (1985). The difficulty of test items that measure more than one ability. Appl Psychol Meas.

[11] R Development Core Team (2018). R: a language and environment for statistical computing [Internet]. http://www.R-project.org.

[12] Robitzsch A (2020). Sirt: supplementary item response theory models: R package version 3.9-4 [Internet]. https://CRAN.R-project.org/package=sirt.

[13] Waugh RF, Chapman ES (2005). An analysis of dimensionality using factor analysis (true-score theory) and Rasch measurement: what is the difference?: which method is better?. J Appl Meas.

[14] Kline RB (2011). Principles and practice of structural equation modeling.

[15] Zhang J, Stout W (1999). The theoretical DETECT index of dimensionality and its application to approximate simple structure. Psychometrika.

[16] Seo DG, Choi Y, Huh S (2017). Usefulness of the DETECT program for assessing the internal structure of dimensionality in simulated data and results of the Korean nursing licensing examination. J Educ Eval Health Prof.

